# Investigating the Accuracy of Quantitative Echocardiographic-Modified Task Force Criteria for Arrhythmogenic Ventricular Cardiomyopathy in Adolescent Male Elite Athletes

**DOI:** 10.1007/s00246-021-02744-5

**Published:** 2021-10-23

**Authors:** Chetanya Sharma, Dan M. Dorobantu, Diane Ryding, Dave Perry, Steven R. McNally, A. Graham Stuart, Craig A. Williams, Guido E. Pieles

**Affiliations:** 1 Bristol Congenital Heart Centre, The Bristol Heart Institute, University Hospitals Bristol NHS Foundation Trust, Upper Maudlin Street, Bristol, BS2 8BJ UK; 2 Children’s Health and Exercise Research Centre, University of Exeter, St Luke’s Campus, Heavitree Road, Exeter, EX1 2LU UK; 3 National Institute for Health Research (NIHR) Cardiovascular Biomedical Research Centre, Bristol Heart Institute, Upper Maudlin Street, Bristol, BS2 8BJ UK; 4 Manchester United Football Club, Football Medicine & Science Department, AON Training Complex, Birch Road, Carrington, Manchester, M31 4BH UK; 5 Institute for Sport Exercise and Health (ISEH), University College Hospital London, London, UK

**Keywords:** Athletes, Adolescents, Cardiomyopathy, Echocardiography

## Abstract

Athlete preparticipation screening focuses on preventing sudden cardiac death (SCD) by detecting diseases such as arrhythmogenic ventricular cardiomyopathy (AVC), which affects primarily the right ventricular myocardium. Diagnosis may be obscured by physiological remodeling of the athlete heart. Healthy athletes may meet the 2010 Task Force Criteria right ventricular outflow tract (RVOT) dimension cut-offs, questioning the suitability of the modified Task Force Criteria (mTFC) in adolescent athletes. In this study, 67 male adolescent footballers undergoing preparticipation screening were reviewed. All athletes underwent a screening for resting ECG and echocardiogram according to the English FA protocol, as well as cardiopulmonary exercise testing, stress ECG, and exercise echocardiography. Athletes’ right ventricular outflow tract (RVOT) that met the major AVC diagnostic criteria for dilatation were identified. Of 67 evaluated athletes, 7 had RVOT dilatation that met the major criteria, all in the long axis parasternal view measurement. All had normal right ventricular systolic function, including normal free-wall longitudinal strain (ranging from − 21.5 to − 32.7%). Left ventricular ejection fraction ranged from 52 to 67%, without evidence of structural changes. Resting ECGs and cardiopulmonary exercise tests were normal in all individuals. In a series of healthy athletes meeting the major AVC diagnostic criteria for RVOT dilatation, none had any other pathological changes on a detailed screening including ECG, exercise testing, and echocardiography. This report highlights that current AVC echocardiographic diagnosis criteria have limitations in this population.

## Introduction

Arrhythmogenic ventricular cardiomyopathy (AVC, also known as arrhythmogenic right ventricular cardiomyopathy, ARVC) is a rare inherited disease characterized by fibrofatty infiltration of the right ventricular myocardium [1]. Presentation can vary from exercise-induced palpitations to congestive cardiac failure. However, a significant minority present with sudden cardiac death (SCD) is the first symptom [2]. Although the disease usually presents in young adulthood, 10% of SCDs from AVC occur in children and adolescents [3], and AVC is responsible for at least 20% of all SCDs in children and adolescents [4]. Athletes with underlying cardiac disease are at greater risk of SCD compared with non-athletic individuals, by virtue of repetitive intense training acting as a trigger for cardiac arrest [5]. There is also a significant male preponderance, with rates in males being 2–25 times higher than females, although the reasons are not fully understood [5, 6]. This increased risk of SCD forms the rationale behind preparticipation screening, as disqualification of affected athletes increases the chance of preventing on-field SCD.

AVC presents a considerable diagnostic challenge as there is no single gold standard method; instead, diagnosis requires a multiparametric approach, as laid out in the modified Task Force Criteria (mTFC) [1] and recently the Padua criteria [7]. In athletes, physiological adaptations to training can obscure and confound the clinical presentation. Ventricular dilatation, mild systolic impairment, and anterior precordial T-wave inversion are well characterized consequences of exercise-induced cardiac remodeling in adults [8–10]. Right ventricular dilatation is a shared characteristic between athletes and AVC. RVOT diameter and RV volume, used in the mTFC and Padua criteria, respectively, may lack specificity in the athlete population. Reference ranges have been proposed for athletes to help differentiate physiological conditioning from AVC but only in adults [11–14]. However, children and adolescents with AVC are less likely to have developed significant structural, functional, and electrical alterations. Both the mTFC and Padua criteria are based almost entirely on non-athletic adult data, and there still remains a lack of pediatric-specific guidance despite acknowledgment that current criteria are inadequate for this group [15]. It has recently been shown that current echocardiographic mTFCs are not met by the majority of adolescent AVC patients [16].

The aim of this study was to assess a group of male adolescent footballers and subsequently report on those with significant right ventricular outflow tract (RVOT) dilatation meeting the size criteria proposed in the mTFC. It was hypothesized that athletes, despite a dilated RVOT, will display no other evidence of cardiac structural or functional abnormality when assessed by conventional 2-dimensional echocardiography, speckle-tracking echocardiography (STE), resting electrocardiogram or cardiopulmonary exercise testing (CPET).

## Methods

Data for male adolescent athletes from an English Premier League football academy undergoing preparticipation screening between 2015 and 2017 were reviewed. All athletes performed more than 10 h training per week. As part of a prospective case series study, all players signed an assent form, with accompanying parent/guardian consent. National Institute for Health Research (NIHR) ethics board approval was obtained. Participants underwent an evaluation protocol on a single day in the same test facility by two clinicians (GP and CAW). Participants were screened for cardiac disease with a preparticipation questionnaire, physical examination and medical history, 12-lead ECG, resting echocardiography, and exercise echocardiography with cardiopulmonary exercise testing.

**Fig. 1:**
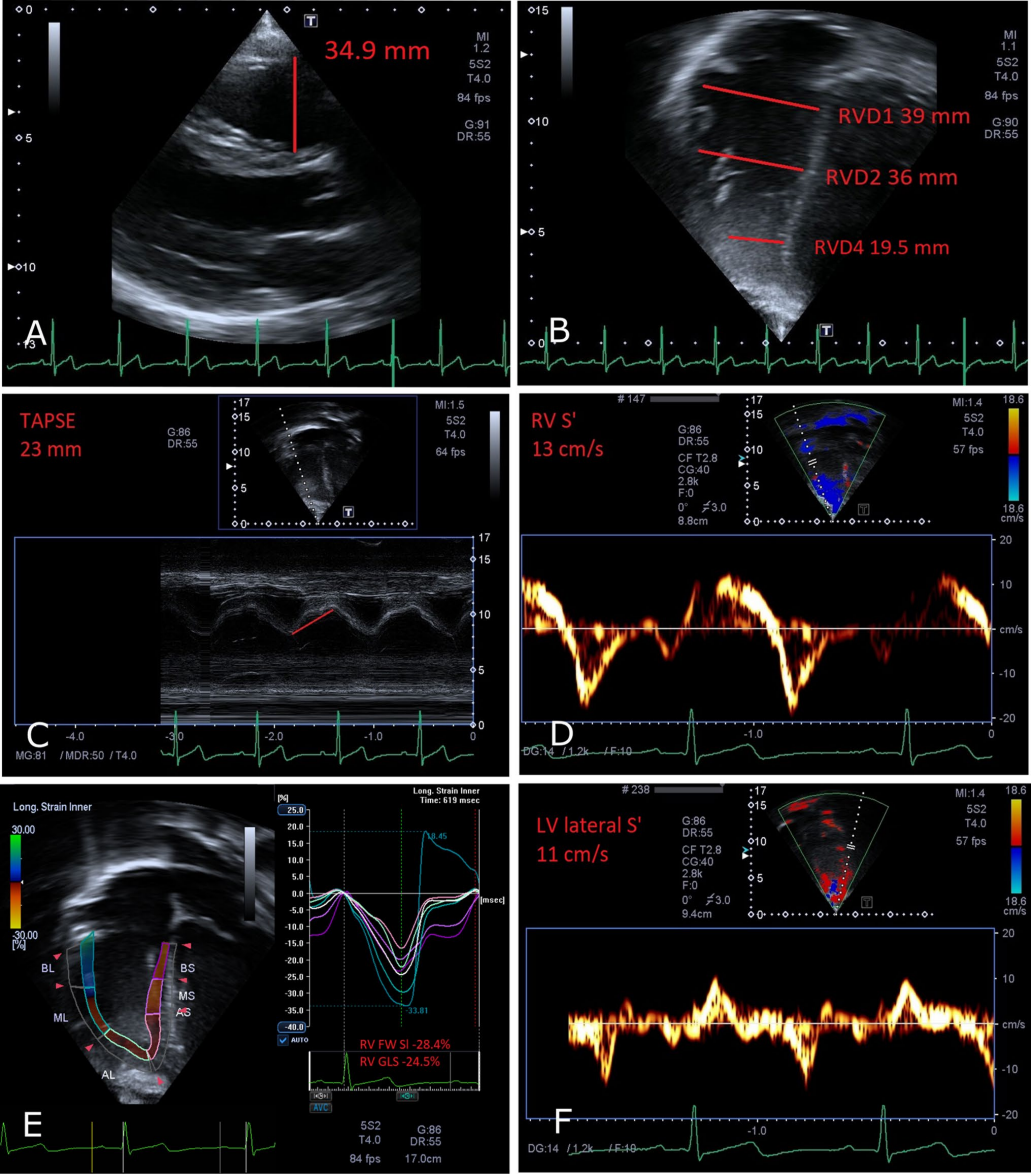
Echocardiographic biventricular size and systolic function measurements in Athlete 1. **A** PLAX view showing RVOT end-diastolic diameter. **B** A4C view showing RV basal (RVD_1_), mid (RVD_2_), and apical (RVD_4_) end-diastolic diameters. **C** M-mode view showing TAPSE. **D** Tissue Doppler imaging showing the peak systolic tricuspid annular velocity (RV S’). **E** Speckle-tracking echocardiography showing RV segmental strain curves, free-wall RV strain (RV Sl), and global RV strain (RV GLS). **F** Tissue Doppler imaging showing the peak systolic mitral lateral annular velocity (LV lateral S’)

**Fig. 2:**
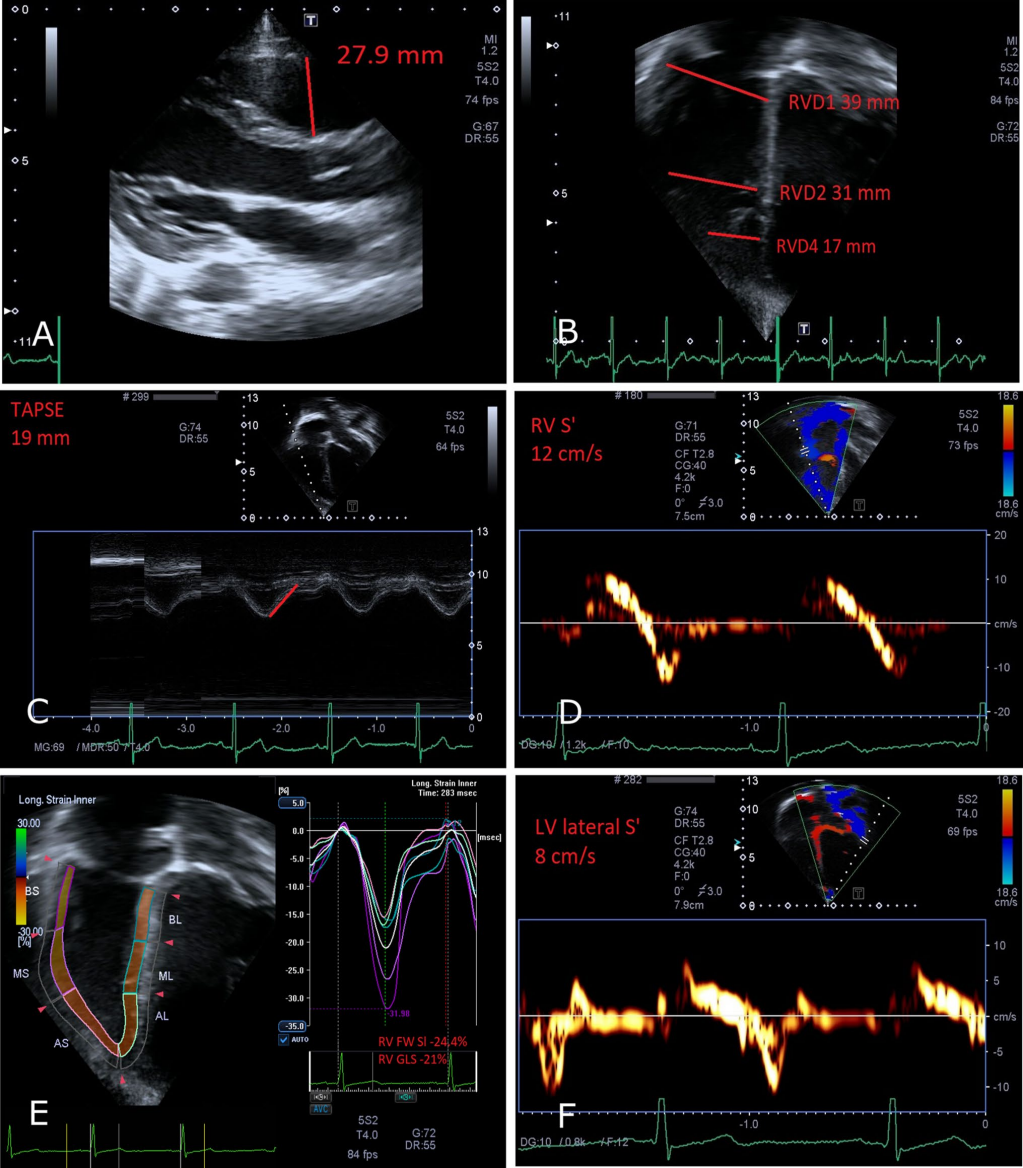
Echocardiographic biventricular size and systolic function measurements in Athlete 3. Panel descriptions as for Fig. 1

An incremental CPET on a recumbent cycle ergometer (Ergosana GMBH, Bitz, Germany) positioned at a 45° inclination (25 W·3/min increments) was performed to volitional exhaustion at a pedaling frequency of 70 ± 5 rpm. Echocardiographic measurements were performed in supine position and analysis performed using an Artida machine, 2.0–4.8 MHz transducer, and UltraExtendV3.2 software (Canon Medical Systems, Japan). Measurements of 2-dimensional, tissue Doppler (TDI) and pulsed-wave (PW) Doppler were recorded in the clinical report and are reproduced in Figs. 1 and 2, for readability only. For the RVOT diameters, one clinician assessed measurements at two different times, and a second clinician (DMD) also assessed the sample. Inter- and intra-observer reliability of RVOT measurements were reported as intraclass coefficient. Speckle-tracking analysis was conducted offline by one clinician (DMD). When warranted, further clinical examinations were recommended based on the local protocol, with no confirmed cardiomyopathy case being reported.

End-diastolic RV diameters were measured in the parasternal long-axis view of the RVOT, proximal parasternal short-axis view of the RVOT, and distal parasternal short-axis view of the RVOT. Apical views were used to measure the end-diastolic transverse diameter at the RV base (RVD_1_), mid-diameter (RVD_2_), base-to-apex length (RVD_3_), and apical diameter (RVD_4)_ [16]. All chamber size measurements were performed in accordance with the current guidelines [17]. RV function was quantified using tricuspid annular peak systolic excursion, RV FAC %, and PW TDI at the lateral tricuspid annulus [18, 19].

For this case series athletes meeting, the major mTFC criteria for RVOT dilatation (long axis RVOT ≥ 32 mm or ≥ 19 mm/m^2^; short axis proximal RVOT ≥ 36 mm or ≥ 21 mm/m^2^) were selected [1]. Right ventricle (RV) volume data were not available at this point, so global RV dilatation Padua Criteria could not be used [7]. RV and LV size, RV function, and LV function age/body size normative values were used to determine abnormal values, in addition to current adult-derived recommendations [17, 20–26]. A left ventricular ejection fraction (LVEF) of less than 55% was considered abnormal [27].

## Results

Of the 67 athletes evaluated, 7 met the major mTFC for RVOT dilatation (age range 13.4–15.9 years). RVOT sizes for each patient are detailed in Table 1. All 7 had dilated RVOT measured in the parasternal long-axis view (RVOT_PLAX_), either absolute (*n* = 5) or indexed (*n* = 2), but none met the proximal RVOT dilatation criteria in parasternal short-axis view (RVOT_PSAX-proximal_) or had a distal RVOT measured in parasternal short-axis view of > 27 mm. Three athletes had an abnormal/borderline echocardiographic parameter: Athlete 1, Athlete 3, and Athlete 6. The main echocardiographic findings for athletes 1 and 3 are represented in Figs. 1 and 2, respectively.

**Table 1:** Individual patient demographics and RVOT measurements

Participant	Age (y)	Stature (cm)	Body mass (kg)	BSA (m^2^)	RVOT_PLAX_ (mm)	RVOT_PLAX_/BSA (mm/m^2^)	RVOT_PSAX—proximal_ (mm)	RVOT_PSAX-proximal_/BSA (mm/m^2^)	RVOT_PSAX-distal_ (mm)
Athlete 1	15.9	177.4	71.7	1.9	34.9	18.5	33.1	17.6	NR
Athlete 2	16.0	178.3	60.5	1.76	32.5	18.5	34.3	19.5	22.3
Athlete 3	14.3	157.9	44.8	1.4	27.9	19.7	26.6	18.7	15.8
Athlete 4	15.2	173.8	64.2	1.8	32.3	18.2	33.3	18.8	21.7
Athlete 5	13.4	165.1	48.3	1.51	29.7	19.6	28.9	19.1	20.3
Athlete 6	15.7	179.5	74	1.93	32.7	17.0	33.2	17.2	22.1
Athlete 7	15.7	171.2	64.5	1.76	32.6	18.6	28.2	16	21.6

Intra-observer reliability, ICC: RVOT PLAX = 0.73, RVOT SAX1 = 0.94, RVOT SAX2 = 0.84

Inter-observer reliability, ICC: RVOT PLAX = 0.77, RVOT SAX1 = 0.88, RVOT SAX2 = 0.60 *BSA* body surface area, *PLAX* parasternal long axis, *PSAX* parasternal short axis, *RVOT* right ventricular outflow tract

Intra-observer reliability, ICC: RVOT PLAX = 0.73, RVOT SAX1 = 0.94, RVOT SAX2 = 0.84

Inter-observer reliability, ICC: RVOT PLAX = 0.77, RVOT SAX1 = 0.88, RVOT SAX2 = 0.60

*BSA* body surface area, *PLAX* parasternal long axis, *PSAX* parasternal short axis, *RVOT* right ventricular outflow tract

Table 2 details additional RV size and systolic function parameters. One athlete (Athlete 1, Fig. 1) showed a borderline-enlarged RV mid-inflow diameter (RVD_2_). One athlete (Athlete 6, Fig. 2) showed borderline, but normal for age, RV peak systolic velocity by TDI. FAC was normal for all athletes. No athletes showed abnormal RV free-wall strain.

**Table 2:** Individual participant RV parameters

Participant	RVD_1_ (mm)	RVD_2_ (mm)	RVD_3_ (mm)	RVD_4_ (mm)	RVS’ (cm/s)	TAPSE (mm)	FAC (%)	Free wall strain (%)
Athlete 1	39	36	71	19.5	13	23	54	28.4
Athlete 2	37	28	65	18.5	15	34	56	32.7
Athlete 3	39	31	67	17.0	12	19	45	24.4
Athlete 4	35	25	59	18.5	15	NR	63	24.6
Athlete 5	42	35	85	19.2	14	19.9	50	26.2
Athlete 6	40	30	77	19.8	13	14.3	43	21.5
Athlete 7	39	22	75	22.2	12	23	60	27.0

*FAC* fractional area change, *RV* right ventricle, *RVD*_*1*_ right ventricle basal-inflow diameter, *RVD*_*2*_ right ventricle mid-inflow diameter, *RVD*_*3*_ right ventricle base-to-apex length, *RVD*_*4*_ right ventricle apical-third diameter, *RVS’* systolic tricuspid annular velocity, *TAPSE* tricuspid annular plane systolic excursion

The LV size and function parameters are shown in Table 3. Only one athlete (Athlete 3) had borderline-low left ventricular ejection fraction and LV peak systolic velocity by TDI. All other LV parameters, including apical-4-chamber strain, were within the normal range for all athletes.

**Table 3:** Individual LV parameters

Participant	IVS thickness (cm)	PW thickness (cm)	EDD (cm)	ESD (cm)	EF (%)	LV S’ (cm/s)	E’ (cm/s)	A4C strain
Athlete 1	1.1	0.9	4.5	3.2	61	11	14	NR
Athlete 2	1.1	1.1	4.8	3.4	61	11	17	19.86
Athlete 3	0.9	0.8	4.3	3.2	52	8	10	NR
Athlete 4	0.9	1.1	4.3	3.0	60	NR	NR	NR
Athlete 5	1	0.7	3.9	2.4	59	10	16	20.26
Athlete 6	1.1	1	4.6	3.3	67	11.5	12	NR
Athlete 7	1.1	1	4.9	3.9	56	10	17	19.32

*A4C* apical-4-chamber, *EDD* end-diastolic diameter, *EF* ejection fraction, *ESD* end-systolic diameter, *IVS* interventricular septum, *LV* left ventricle, *NR* not recorded, *PW* posterior wall

All athletes had a normal resting 12-lead ECG, normal cardiopulmonary exercise test (CPET) values of a trained athlete and normal exercise echocardiogram.

## Discussion

This study assessed reported detailed clinical, imaging, and physiological data on adolescent athletes with significant right ventricular outflow tract (RVOT) dilatation meeting the 2010 mTFC [1]. There were no significant echocardiographic (including speckle tracking), ECG, CPET, or exercise echocardiographic findings suggestive of pathology, except for the RVOT dilation. Athletes had no relevant symptomatology, family history, or past medical history. These observations show the need to re-evaluate how RVOT dilatation is interpreted for the diagnosis of AVC in adolescent athletes. At present, there is a paucity of research on the subject of using the mTFC for AVC in the pediatric athlete population, against a background of increasing participation of children and adolescents in competitive sport [28]. In this unique patient population, two challenges in diagnosis arise: differentiating pathology from physiology of the athlete heart and the need to use criteria designed for adults in a pediatric setting.

In our sample of 67 athletes, 7 athletes met the structural RVOT_PLAX_ criteria. No athletes from our sample had a RVOT_PSAX_ that met the major mTFC RVOT cut-off. This is in-keeping with a systematic review and meta-analysis by D’Ascenzi et al. (2017) on normative right heart values in adult athletes, which found that male athletes had higher upper limits for RVOT_PLAX_, while RVOT_PSAX_ values were comparable to controls [13].

It is important to note that while the included athletes fulfilled the quantitative major echocardiographic thresholds of the mTFC, they did not display the prerequisite regional RV akinesia, dyskinesia, or aneurysm required for the mTFC echocardiographic major criterion. However, in practice, clinicians cannot rely on these morphological abnormalities given that most children would be expected to be in the early stages of disease. Furthermore, visual assessment of these functional abnormalities may be challenging in young athletes exhibiting significant RV remodeling [29]. In comparison with cardiac magnetic resonance imaging (CMR), echocardiography may be insensitive to early and subtle changes due to limitations in acoustic windows to the RV free wall [30]. Indeed, one study showed that only 50% of patients with CMR-positive AVC fulfilled echocardiographic mTFC [31]. Therefore, clinicians should not expect adolescents to exhibit the RV wall motion abnormalities that are the hallmark of AVC, representing a further limitation of both the mTFC and the Padua criteria in adolescents. Crucially, this leads to an inevitable increased reliance on RVOT dimensions to guide any suspicion of diagnosis during preparticipation screening, which, as shown in this study, are not appropriate in the adolescent athlete population and might lead to unnecessary investigations, delays in treatment, and patient anxiety. In addition, while diagnostic criteria encourage scaling of RVOT dimensions to body surface area (BSA), this is not always helpful in differentiating AVC from controls [16] and allometric scaling of RV chamber size to BSA may better ensure size independence [11].

Novel techniques such as speckle-tracking echocardiography have been shown to be useful discriminators in adults and adolescents with AVC and help in the early diagnosis [16, 32]. In a recent study, RV longitudinal strain and the apical RV end-diastolic diameter (RVD_4_), which was, therefore, also included in our analysis (Table 2), were abnormal in adolescent AVC patients and predictors of disease, despite not meeting the mTFC for RVOT dilatation [16]. These data also support our finding that the mTFCs have a low diagnostic sensitivity in adolescents. Furthermore, using RV strain measurements may not only increase the diagnostic yield of echocardiography in AVC to diagnose but also exclude the disease, as shown in our cohort.

The recently proposed Padua criteria replace RVOT diameter with end-diastolic volumes in the morpho-functional category for AVC diagnosis. While these useful parameters are readily obtained by modern CMR, they are challenging to obtain using echocardiography and, while possible, are not part of standard assessment [33]. As in the mTFC, there persists the prerequisite for RV akinesia, dyskinesia, or bulging, with its aforementioned limitations in the pediatric population as they are rarely found using echocardiography in adolescents with AVC [16, 30].

The limitations of this case study design are acknowledged. No statistical comparisons were made with non-athletic adolescents or the adolescent athletes not meeting the mTFC for RVOT dilatation. Instead, this study sought to explore individual case data rather than aggregate results, so that granular, detailed data on each athlete could be presented. We also did not explore other mTFC parameters such as cardiac magnetic resonance imaging, family history, and genetics. Furthermore, only male football players were included, thus, not representing a truly diverse athlete population with female players and other sporting disciplines.

In the future, new techniques such as tissue Doppler and speckle-tracking imaging should be explored in preparticipation screening, especially in cases of abnormal structural changes such as RVOT dilatation. Challenges in implementing these techniques include a lack of data-acquisition protocols and normative data for adolescent athletes, as well as operator experience with STE analysis. Furthermore, routine preparticipation echocardiography does imply an increase on resource allocation, from experienced clinical staff to increased costs, and for these reasons, its implementation is still sparse.

In summary, it was shown in this adolescent athlete population that a dilated RVOT in isolation should not be viewed as a ‘red flag’ for the diagnosis of AVC. The results from this case series indicate the need to re-evaluate the appropriateness of current echocardiographic criteria for AVC diagnosis in adolescent athletes. While the limitations of echocardiography in the diagnosis of AVC are acknowledged, echocardiography is the primary imaging modality in cardiac screening of athletes, and therefore, more accurate screening protocols for AVC in this population are needed.
